# ﻿A new millipede genus and species of the tribe Pachyiulini from the Caucasus (Diplopoda, Julida, Julidae)

**DOI:** 10.3897/zookeys.1097.81792

**Published:** 2022-04-19

**Authors:** Aleksandr P. Evsyukov, Boyan Vagalinski, Igor Y. Zabiyaka, Evgeniy V. Sadyrin

**Affiliations:** 1 Don State Technical University, Department of Biology and General Pathology, Rostov-on-Don, Russia; 2 Institute of Biodiversity and Ecosystem Research at the Bulgarian Academy of Sciences, Sofia, Bulgaria; 3 Don State Technical University, Research and Education Center “Materials”, Rostov-on-Don, Russia

**Keywords:** Azerbaijan, *Bellatoiulusgolovatchi*, cybertype, taxonomy

## Abstract

A new genus and species of the millipede tribe Pachyiulini, *Bellatoiulusgolovatchi***gen. et sp. nov.**, is described from the Lesser Caucasus, Azerbaijan. Cybertypes of the new species are created from the physical holotype male and from a paratype female. The distribution and ecological features of the new species, and the position of the new genus within Pachyiulini are discussed.

## ﻿Introduction

The tribe Pachyiulini, also referred as the subfamily Pachyiulinae, is a monophyletic trans-Palaearctic group (Enghoff et al. 2013), but most of its diversity is restricted to the Mediterranean Region. It includes 15–20 genera (Antić et al. 2018), or 16–22 genera or subgenera (Vagalinski 2020). According to Hoffman (1980), the tribe includes both the largest and the smallest members of Julidae.

In the Caucasus *sensu lato*, the tribe is represented by three genera: *Pachyiulus* Berlese, 1883, *Amblyiulus* Silvestri, 1896, and *Syrioiulus* Verhoeff, 1914 (Evsyukov 2016; Evsyukov et al. 2021). While the largest julid millipedes belong to the former genus, the latter two genera include julids of mostly moderate size. No minute Pachyiulini or “micropachyiulinines”, like for example the genera *Geopachyiulus* Verhoeff, 1899, *Micropachyiulus* Verhoeff, 1899, or *Hylopachyiulus* Attems, 1904, have hitherto been known from the Caucasus.

Here we describe a new genus for a tiny new species of this tribe from the Azerbaijani part of the Lesser Caucasus and create cybertypes of the new species from the physical male holotype and a female paratype. Until now cybertypes have not been very common in millipede taxonomy and are known only for two species (Akkari et al. 2015; Rosenmejer et al. 2021).

## ﻿Materials and methods

The specimens were stored in 70% ethanol. All material has been shared between the collections of the Zoological Museum of the Moscow State University, Russia (**ZMUM**), the National Museum of Natural History, Sofia, Bulgaria (**NMNHS**), and the Natural History Museum of Denmark, Copenhagen, Denmark (**NHMD**) (formerly ZMUC–Zoological Museum, University of Copenhagen). Some body parts of males (antennae, gonopods, legs, etc.) and females (vulvae and leg pairs 2) were dissected and mounted in glycerol on temporary microscopic slides or prepared for scanning electron microscopy (SEM). Photographs were taken using a Zeiss StereoDiscovery V.20 microscope and processed with Zeiss ZEN software (Don State Technical University, Rostov-on-Don, Russia). Line drawings were executed using microphotographs taken with a ProgRes C7 digital camera connected to a Zeiss Axio Imager 2 microscope (Institute of Biodiversity and Ecosystem Research, Sofia, Bulgaria), which were then displayed on a laptop screen and copied on tracing paper.

Scanning electron micrographs were taken with a Zeiss CrossBeam 340 (Don State Technical University, Rostov-on-Don, Russia). For SEM micrographs, the gonopods, legs etc. were dehydrated in alcohol (96 and 100%) and acetone, glued to aluminium SEM-stubs and air dried.

Creation of cybertypes was carried out using a Zeiss Xradia Versa 520 X-ray computed micro-tomography unit (Don State Technical University, Rostov-on-Don, Russia). X-ray projections acquisition parameters were as follows: X-ray tube voltage 60 kV, power 4.5 V, magnification objective 0.4×, sample rotation 360°, exposure time 4 s, X-ray tube filter LE6. For each scan 2001 projections in total were obtained. The source-object-detector distances were adjusted according to the sample sizes to give the desired field view for each sample. Thus, the voxel sizes were 4.7 and 5.8 µm for the male holotype and the female paratype, respectively. For each scanning, the tube with the sample was placed at the closest possible distance to the X-ray source. The 2048 × 2048-pixel CCD camera was kept at –59 °C and the acquisition was performed with camera binning factor = 2, which resulted in up to 1024 × 1024-pixel sized projection images. The X-ray source filter was selected based on the observed transmittance values according to the recommendations of the Xradia Versa 520 User’s Guide A003030 Rev. B. The exposure time was selected to maintain count (intensity) values >5000 with the selected source parameters and filter. The option for Dynamic Ring Removal, which enables small random motions of the sample during acquisition, was enabled for all projections. During each tomography procedure, 10 reference (air) X-ray images were acquired with equal time intervals between them. Average of these references was applied to each projection. An up to one-hour warm-up scan was performed with the same source parameters before each acquisition. X-ray projections were reconstructed using XRMReconstructor 12.0.8086.19558 software with manually adjusted center shift values, σ = 0.5 Gaussian blur filter and BH = 0.05 standard beam hardening correction.

The reconstructed tomographic images were exported as 16-bit DICOM image stacks (without compression and retaining the original voxel values) for volume rendering in VGStudio MAX 3.5 software (Volume Graphics GmbH, Heidelberg, Germany). The DICOM files were rendered using *Volume renderer (Phong)*. The *Draw* instrument was used to highlight individual structures, and after initial highlighting the region of interest was modified using *Smoothing* tool.

For contrasting before micro-CT examination samples were soaked in 100% alcohol for 2 h and then transferred to a 1% iodine alcohol solution for 48 h (Metscher 2009). For stabilisation of the samples during scanning, they were mounted in plastic tubes (1 ml syringe) in 0.3% aqueous agarose (Metscher 2011). Cybertypes were created from the physical male holotype and a female paratype (Faulwetter et al. 2013). All 3D images and metadata of the physical types are deposited at https://morphobank.org (O’Leary and Kaufman 2012), project number 4180. After examination all material was returned to 70% alcohol.

The distribution map was created using Google Earth Pro 7.3.4.

All images were processed in Adobe Photoshop CS6.

### ﻿Descriptive abbreviations and symbols

**BRF** body ring formula. Indicates the number of podous (including collum and the gonopod-bearing segment/ring) and apodous segments/rings in an individual. This formula is **p+a+T**, where **p** is the number of podous body rings, **a** is the number of apodous body rings, and **T** represents the telson (Enghoff et al. 1993). Only adults have been analysed in the present study.

**H** vertical body diameter measured at a mid-body ring

**L** body length measured at the ozopore level

## ﻿Taxonomic part

### ﻿Class Diplopoda Blainville-Gervais, 1844

**Order Julida Leach, 1814**

#### Family Julidae Leach, 1814

##### ﻿Genus Bellatoiulus gen. nov.

Taxon classificationAnimaliaJulidaJulidae

426253DA-7466-5223-8B59-391E6A320CE4

http://zoobank.org/E87D3EDE-5038-439A-A904-7E0F87BB4546

###### Type species.

*Bellatoiulusgolovatchi* gen. et sp. nov., by present designation.

###### Diagnosis.

A genus of the julid tribe Pachyiulini, distinguished from all contribal genera by the unique presence of a lateral process on the opisthomere, as well as by the following combination of gonopodal and external somatic characters: promere with mesal ridge distally extending in a strong process, caudal face without apical denticles/ridges; opisthomere without anterior and caudal lamellae or these being vestigial, mesomeral process well-differentiated, crest-like, solenomere with a deep and narrow apical fovea; ommatidia absent, mandibular stipites in males not expanded, vertigial and metazonal setae present, pre-anal ring with an epiproct.

###### Name.

Derived from the Latin *bellator* meaning “soldier”, “warrior”, after the remarkably “armed” appearance of the gonopods including the club-like mesal and lateral processes of the promere and the opisthomere, respectively, and the apically serrated mesomeral process; plus *Julus*, the type genus of the family Julidae. Masculine.

##### ﻿ Bellatoiulus golovatchi

Taxon classificationAnimaliaJulidaJulidae

gen. et sp. nov.

0D955BFA-370F-5C2D-B82F-8D8EDE4F6E00

http://zoobank.org/A8A39314-2E0B-4E99-A65A-EBB595D16EF2

###### Material examined.

***Holotype***: ♂ (unbroken) (ZMUM): Azerbaijan, Drmbon [Heyvalı] ca 30 km WSW of Mardakert [Ağdərə], 800–850 m a.s.l., *Quercus*, *Carpinus*, *Acer*, etc. forest, litter, 1–2.VI.1987, S. Golovatch & K. Eskov leg. ***Paratypes***: 1 ♂ (in 3 pieces, with dissected gonopods, leg-pair 2, a mid-body and an end-body leg) , 1 ♀ (in 2 pieces, right vulva dissected) (NMNHS), 1 ♂ (in 2 pieces, with dissected leg-pair 2 and a mid-body leg), 1 ♀ (unbroken) (NHMD), 6 ♂♂ (4 unbroken, 2 in 2 pieces, with dissected gonopods, head, antenna, leg-pairs 1, 2, 3, 8 and a mid-body leg), 42 ♀♀ (30 unbroken, 11 broken in two or more pieces, 1 with dissected vulvae and leg-pair 2), 21 juv. (ZMUM), same collecting data as for holotype.

###### Non-type material:

7 ♂♂, 12 ♀♀, 2 juv. (ZMUM), Azerbaijan, Nadirkhanly [Nadirxanlı] ca 12 km NE of Kelbajar [Kəlbəcər], 1200 m a.s.l., *Fraxinus* & *Juglans* stand, litter, 1.VI.1987, S. Golovatch & K. Eskov leg.; 2 ♂♂ (1 ♂ in 2 pieces, with dissected gonopods), 10 ♀♀, 8 juv. (ZMUM), Azerbaijan, ca 15 km WSW of Mardakert [Ağdərə], 1100 m a.s.l., *Quercus*, *Fagus*, *Acer*, etc. forest, litter, 2.VI.1987, S. Golovatch & K. Eskov leg.

###### Cybertypes:

http://morphobank.org/permalink/?P4180. Created from the physical ♂ holotype and a ♀ paratype.

###### Name.

The new species honours our friend and colleague, Prof. Sergei Golovatch (Moscow, Russia), an outstanding myriapodologist and one of the collectors of the material used for the present species description.

###### Description.

Measurements: holotype with BRF 45+2+T, L = 12 mm, H = 0.6 mm; paratype and non-type ♂♂ with BRF 40–46+1–3+T, L = 10–12 mm, H = 0.5–0.6 mm; paratype and non-type ♀♀ with BRF 37–51+1–4+T, L = 9.5–14 mm, H = 0.5–0.7 mm.

**Figure 1.**
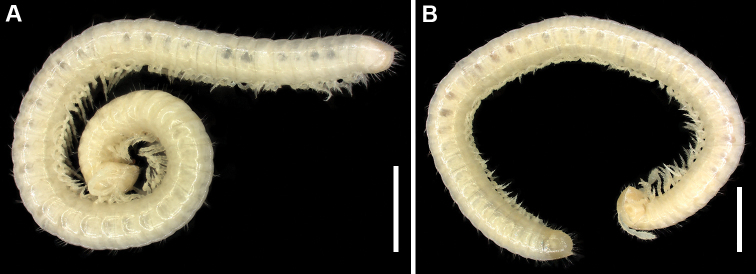
Habitus of *Bellatoiulusgolovatchi* gen. et sp. nov. **A** male paratype **B** female paratype (both ZMUM). Scale bars: 1.0 mm.

Colouration (Fig. 1): completely pallid, gut and defence glands partly visible by transparency (alcohol-fixed material).

**Figure 2.**
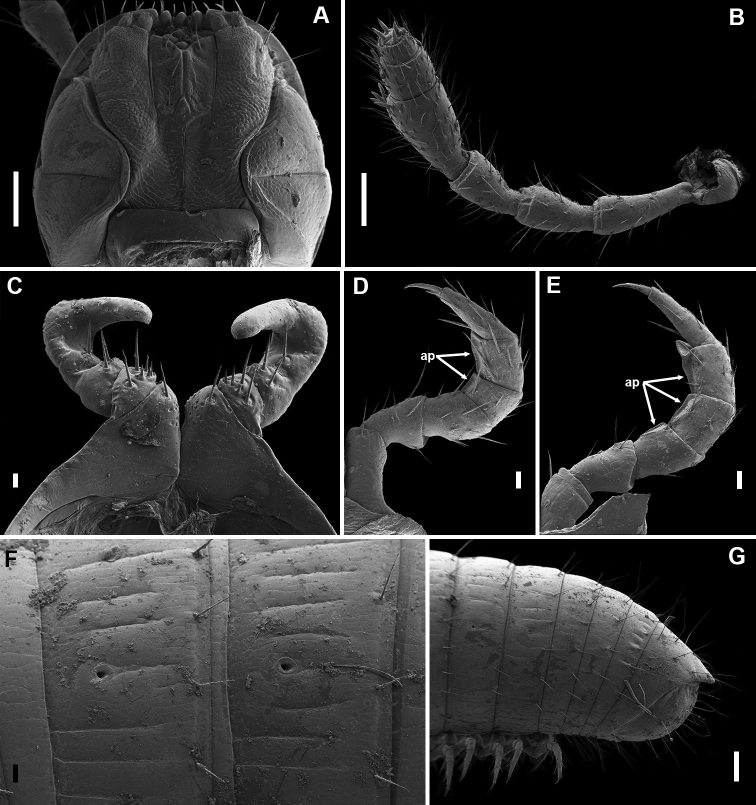
*Bellatoiulusgolovatchi* gen. et sp. nov., male paratype (ZMUM). **A** head, ventral view **B** right antenna, lateral view **C** leg pair 1, frontal view **D** right leg 2, frontal view **E** left leg 3, caudal view **F** ozopores in midbody rings, lateral view **G** posterior part of body, lateral view. Abbreviation: **ap** adhesive pads. Scale bars: 0.1 mm (**A, B, G**); 0.01 mm (**C**); 0.02 mm (**D, E, F**).

Head (Figs 2A, 1, 5, 8): ommatidia absent. 2 vertigial, 4 (usually) or 5 (rarely) supralabral and 8–14 labral setae. Antennae (Fig. 2B) 1.4–1.7 times as long as head in males, and 1.3–1.4 times in females; antennomeres 2 and 5 subequal in length, somewhat longer than 3 and 4, and much longer than 6; 5 ca 1.6 times as long as broad and ca 1.5 times as broad as 2; the four apical cones relatively small; distal margins of antennomeres 5 and 6 dorsolaterally with several bacilliform sensilla basiconica, those on antennomere 5 of similar size to the apical cones, those on antennomere 6 somewhat finer. Mandibular stipites in males not expanded. Labrum tridentate. Gnathochilarium without peculiarities; proximal part of stipites non-setose, distally with three setae usual for the family, stipital palps normally developed, each bearing a group of extremely small apical sensilla; promentum small, rhomboid, separating lamellae linguales in roughly their proximal third, the latter each bearing three or four setae in a longitudinal row.

Trunk and legs: collum mostly smooth, with only two or three faint longitudinal grooves just next to posterolateral corner. Body rings (Figs 1, 2F, G, 5, 8) slightly to moderately vaulted. Prozonae generally smooth, with sparse, very short and fine, irregular striae. Metazonae with sparse, shallow longitudinal striations, most striae not crossing entire length of metazona; hind margins with a rather dense whorl of erect to somewhat slanting setae, these being 0.12–0.2 times as long as H in males, and 0.1–0.17 times in females. Ozopores (Fig. 2F) small, placed behind pro-metazonal suture at ca 1/3–2/5 of metazonal length measured from front to back. Walking legs (Figs 2D, E, 7A) of moderate length: mid-body legs (Fig. 2E) 0.8–0.9 times as long as H in males and 0.7–0.8 times in females. Tarsus of mid-body legs 1.5–1.7 times as long as tibia and ca 3.5 times as long as apical claw. Legs 2 in males with accessory claw, legs 3 and following pairs without; female legs altogether without accessory claw.

Telson (Fig. 2G): Pre-anal ring sparsely covered with long setae. Epiproct short and stout (considerably surpassed by the longest paraproctal setae), straight to slightly bent ventrad, roof-like, ending bluntly without distinct hyaline tip. Hypoproct small, rounded, not protruding behind hind margins of paraprocts in both sexes; ventral surface with a pair of median setae. Paraprocts sparsely to moderately setose, with a row of shorter setae along each paraproct’s caudal margin.

**Figure 3.**
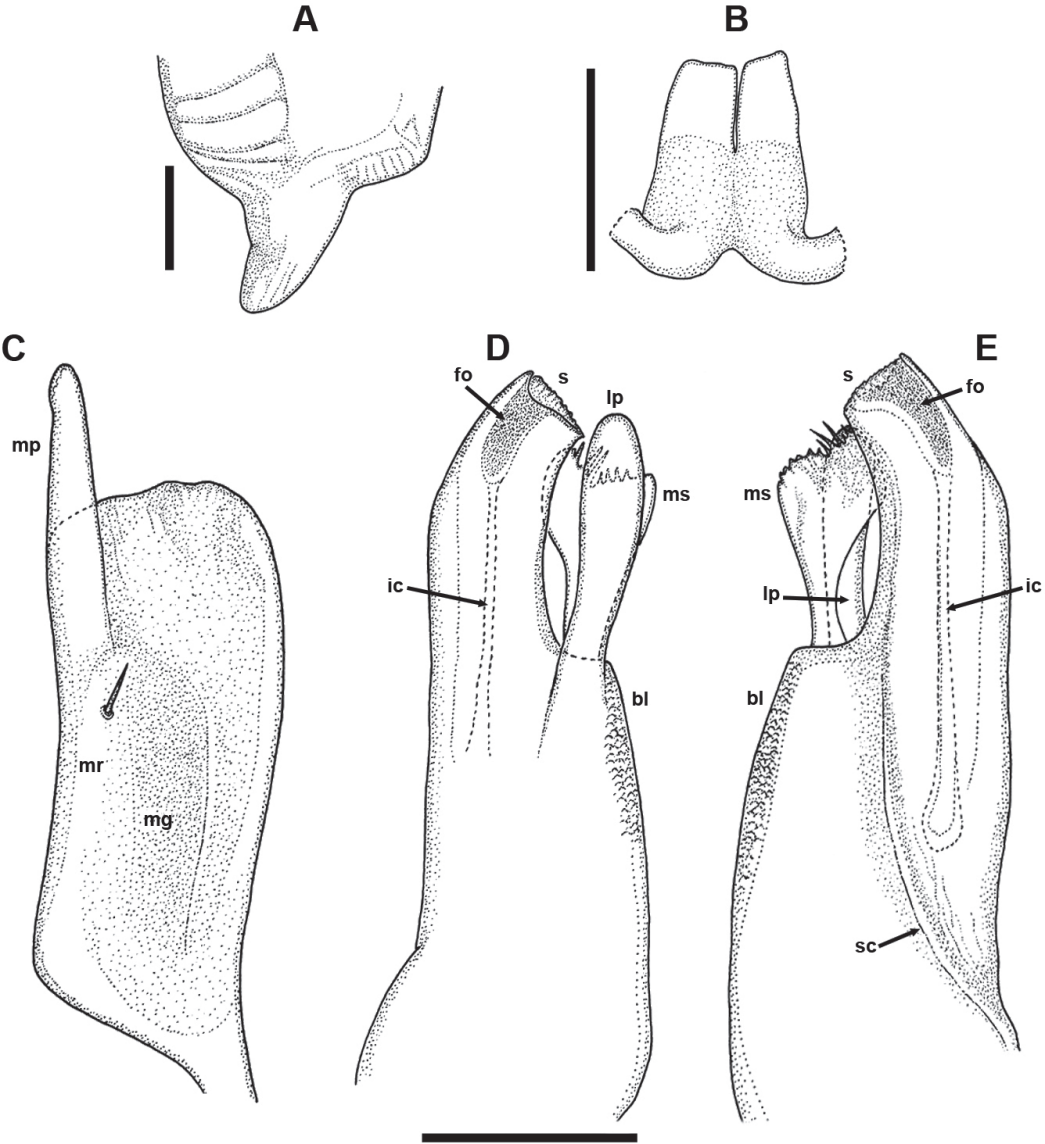
*Bellatoiulusgolovatchi* gen. et sp. nov., male paratype (NMNHS). **A** right ventral edge of pleurotergum 7, ventro-lateral view **B** penis, caudal view **C** left promere, caudal view **D** left opisthomere, lateral, slightly caudal view **E** left opisthomere, mesal view. Abbreviations: **bl** basofrontal lobe **fo** fovea **lp** lateral process **mg** median grove **mp** mesal process **mr** mesal ridge **ms** mesomeral process **ic** (supposed) inner canal **s** solenomere, **sc** sperm canal. Scale bars: 0.1 mm.

Male sexual characters: leg-pair 1 (Fig. 2C) 3-segmented, typical hooks oriented fronto-mesad; tibial outgrowth slender, tarsal remnant absent. Leg-pair 2 (Fig. 2D) slightly thicker than following pairs, ventrally with crested adhesive pads (**ap**), one each on postfemur and tibia; next several pairs (Fig. 2E) with a small femoral pad in addition; the pads gradually diminishing towards end-body, completely disappearing in legs of the last third. Pleurotergum 7 (Fig. 3A) ventrally forming well-pronounced, shovel-like lobes originating from the border zone between pro- and metazona, protruding ventrad behind gonopods, not concealing them from lateral view. Penis (Fig. 3B) very small, non-sclerotised, set deeply above coxae 2, with a short basal part and rather slender, flattened, completely transparent apical lobes.

**Figure 4.**
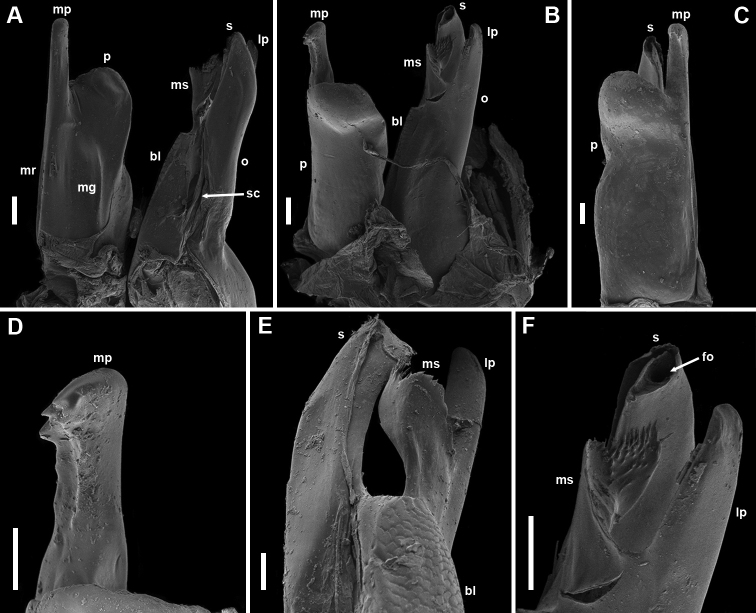
*Bellatoiulusgolovatchi* gen. et sp. nov., male paratype (ZMUM). **A** left gonopods, mesal view (promere strongly turned mesad) **B** right gonopods, fronto-lateral view **C** left gonopods, frontal view **D** mesal process of right promere, fronto-lateral view **E** end of left opisthomere, meso-frontal view **F** end of right opisthomere, fronto-lateral view. Abbreviations: **bl** basofrontal lobe **fo** apical fovea **lp** lateral process **mg** median groove **mp** mesal process **mr** mesal ridge **ms** mesomeral process **o** opisthomere **p** promere **s** solenomere **sc** sperm canal. Scale bars: 0.03 mm (**A, B, F**); 0.02 mm (**C, D, E**).

**Figure 5.**
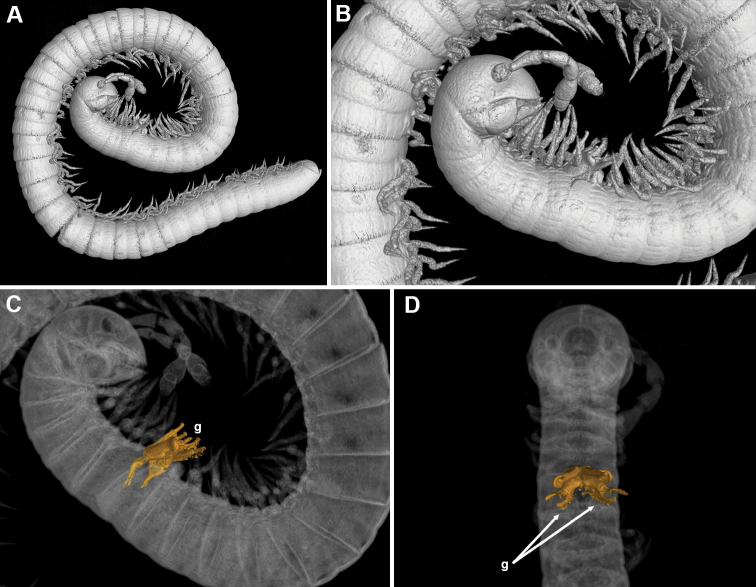
*Bellatoiulusgolovatchi* gen. et sp. nov., male cybertype. **A** habitus, lateral view **B** anterior half of body, lateral view **C** anterior half of body with highlighted gonopods, lateral view **D** anterior body part with highlighted gonopods, ventral view. Abbreviation: **g** gonopods. Images not to scale.

**Figure 6.**
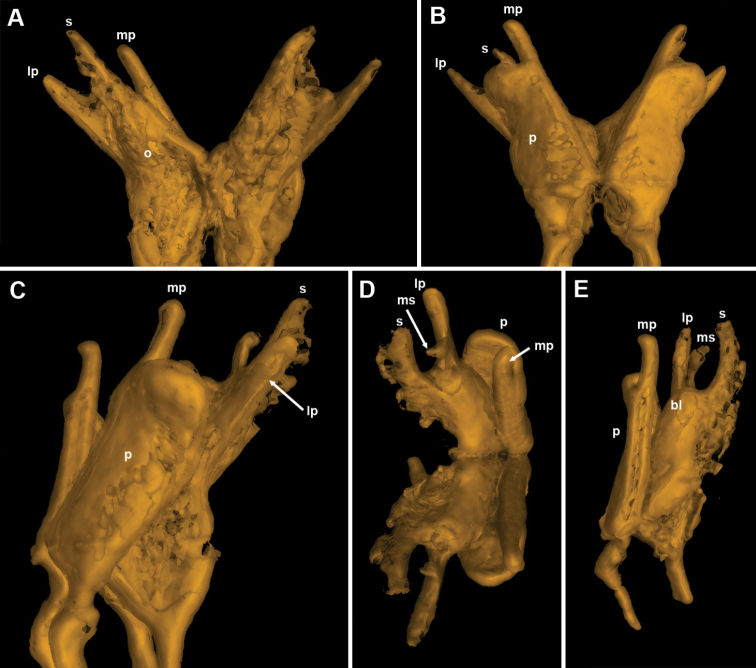
*Bellatoiulusgolovatchi* gen. et sp. nov., male cybertype. **A** gonopods, caudal view **B** gonopods, frontal view **C** gonopods, lateral view **D** gonopods, ventral view **E** left gonopod, mesal view. Abbreviations: **bl** basofrontal lobe **lp** lateral process **mp** mesal process **ms** mesomeral process **o** opisthomere **p** promere **s** solenomere. Images not to scale.

Gonopods (Figs 3C–E, 4, 6, and **g** in Fig. 5C, D): *in situ* protruding from gonopodal sinus with their apical parts. Promere (Fig. 3C, and **p** in Figs 4A–C, 6B–E) rather stout, roughly quadrangular, insignificantly widening distally, with slightly concave mesal margin and gently sigmoid lateral margin, connected in a flat to broadly rounded apical margin; caudal face with a broad median groove (**mg**), and a broad, not too strongly pronounced mesal ridge (**mr**) bearing one or two short setae at mid-height, distally extending in a long and straight mesal process (Fig. 4D, and **mp** in Figs 3C, 4A–C, 6), with a somewhat clavate apex bearing several blunt teeth on its frontal side. Opisthomere (Figs 3D, E, 4E, F, and **o** in Figs 4A, B, 6A, C–E) relatively slender, reaching nearly level to mesal process of promere; frontal face with a broad, massive, microsquamous, basofrontal lobe (**bl**); mesomeral process (**ms**) originating just distally to the lobe, being strongly flattened, somewhat twisted around its axis, distally widening, apically bearing a group of minute seti- or spiniform filaments; a well-developed club or ping-pong paddle-like lateral process (**lp**) with smooth surface, almost reaching level to solenomere; solenomere (**s**) tubular, reaching higher than mesomeral process, distally somewhat bent latero-frontad, with a deep and narrow apical fovea (**fo**), and an (presumably) inner canal (**ic**) running vertically right basally to fovea, mesal side with a sperm canal (**sc**), distally somewhat deviating frontad, caudal lamella absent.

**Figure 7.**
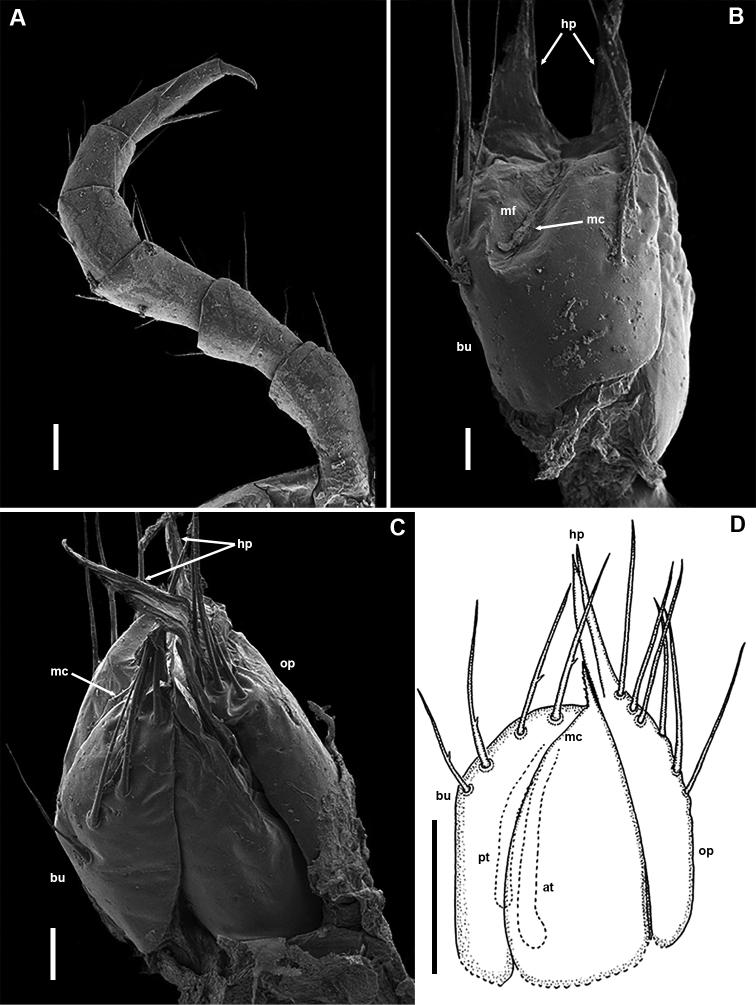
*Bellatoiulusgolovatchi* gen. et sp. nov., female paratypes (ZMUM) **A–C** and NMNHS**D**). **A** right leg 2, caudal view **B** left vulva, caudal view **C** right vulva, fronto-mesal, slightly apical view **D** right vulva, fronto-mesal view. Abbreviations: **at** anterior tube **bu** bursa **hp** hyaline protrusions **mc** median cleft **mf** median field **op** operculum **pt** posterior tube. Scale bars: 0.03 mm (**A**); 0.02 mm (**B, C**); 0.1 mm (**D**).

**Figure 8.**
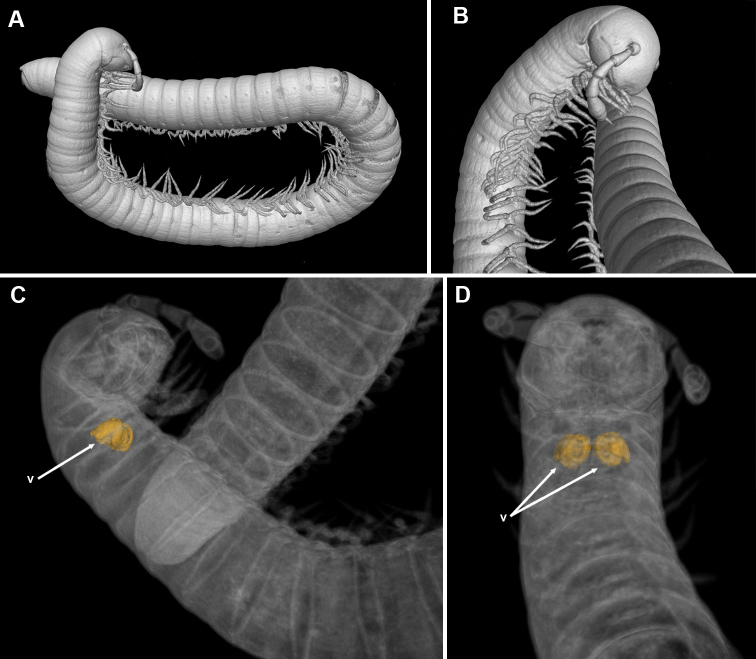
*Bellatoiulusgolovatchi* gen. et sp. nov., female cybertype. **A** habitus, lateral view **B** anterior and posterior body parts, ventrolateral and dorso-lateral views, respectively **C** anterior and posterior body parts with highlighted vulvae, lateral views **D** anterior body part with highlighted vulvae, ventral view. Abbreviation: **v** vulvae. Images not to scale.

**Figure 9.**
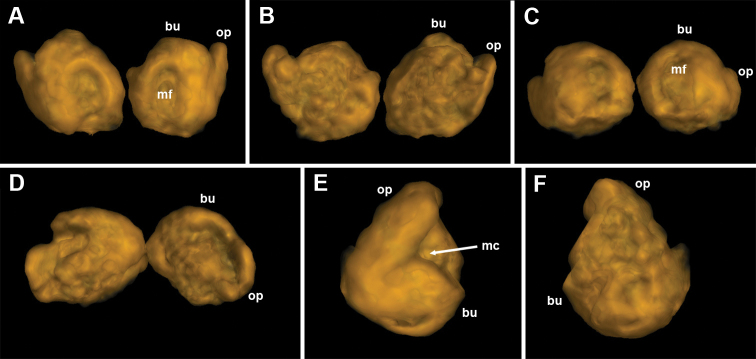
*Bellatoiulusgolovatchi* gen. et sp. nov., female cybertype. **A** vulvae, caudal view **B** vulvae, frontal view **C** vulvae, ventral view **D** vulvae, dorsal view **E** right vulva, lateral view **D** right vulva, mesal view. Abbreviations: **bu** bursa **mc** median cleft **mf** median field **op** operculum. Images not to scale.

Female sexual characters: leg-pairs 1 (Fig. 7A) and 2 only slightly thicker than following legs. Vulva (Figs 7B–D, 9, and **v** in Fig. 8C, D) stout, roughly cylindrical, slightly compressed on sides; bursa (**bu**) mostly symmetric, median cleft (**mc**) positioned on top within a rather deep and narrow median field (**mf**); operculum (**op**) higher than bursa, ending with two very long, tapering, hyaline protrusions (**hp**); a row of four or five setae on each bursal valve and a group of several setae distally on each side of the operculum; bursal side sclerites non-setose. Receptaculum seminis composed of two nearly straight tubes: a shorter and somewhat thinner posterior one (**pt**), and an anterior one (**at**), slightly widening at bottom.

## ﻿Discussion

### ﻿Notes on the position of the genus *Bellatoiulus* gen. nov. within Pachyiulinae/Pachyiulini

*Bellatoiulusgolovatchi* gen. et sp. nov. exhibits a highly distinctive genital (both gonopodal and vulval) morphology, making it hardly comparable to any other genus of Julidae. However, its affiliation to the tribe Pachyiulini seems unquestionable, mainly because of the following male sexual characters: promere without flagellum and with a mesal ridge bearing one or two setae; opisthomere with a well-developed, but not freely articulated, mesomeral process, and with an apical fovea; penis completely non-sclerotised, with relatively long, parallel, apical lobes, without differentiated membranous tubes (corresponding to the “pachyiuline penis type”, as defined by Enghoff (1996)).

The classification of the Pachyiulini is still problematic in terms of both delimitations between genera, and the recognition of natural groups of genera based on synapomorphies. One of the few attempts of a subdivision of the tribe is that of Mauriès (1982), where all genera are divided into three groups depending on the presence/absence of an apical fovea and a flagelloid process on the solenomere. Each of these groups is divided into subgroups based on the presence/absence of a mesomeral process and of some external characteristics, such as vertigial setae, modified male mandibles, etc. The genus *Bellatoiulus* gen. nov. falls into the second group, which is characterized by a well-developed fovea and the absence of a flagelloid process. In addition to the genera *Mesoiulus* Berlese, 1886 (although in fact the type species, *M.paradoxus* Berlese, 1886, lacks a true fovea, as revealed by Enghoff (1992)) and *Cypriopachyiulus* Strasser, 1967, which was recognised by Mauriès (1982), the genera *Parapachyiulus* Golovatch, 1979, *Dangaraiulus* Golovatch, 1979, Caucasian species of *Amblyiulus* Silvestri, 1896, and perhaps *Syrioiulus* Verhoeff, 1914 (the fovea in many of its species is rather shallow, saddle-like) also belong to this group (Golovatch 1979, 2018; Antić et al. 2018; Evsyukov et al. 2021). *Bellatoiulusgolovatchi* gen. et sp. nov. combines plesiomorphic and apomorphic characters according to Mauriès (1982). A plesiomorphic character is the presence of a mesomeral process (shared with all of the above-listed genera). However, other characters are apomorphic. Such are the unmodified male mandibles found in certain species of *Amblyiulus*, *Mesoiulus*, and *Syrioiulus* and the absence of ocelli, as in *Mesoiulus* and in some species of *Amblyiulus* and *Syrioiulus*. Absence of vertigial setae also brings the genus *Bellatoiulus* gen. nov. close to some species of *Amblyiulus*, *Syrioiulus*, *Parapachyiulus*, and *Mesoiulus*.

The new genus differs substantially from other tiny pachyiulinine genera (H of males ca 0.5 mm), such as the Carpathian–Balkan *Hylopachyiulus* Attems, 1904, *Geopachyiulus* Verhoeff, 1899, and *Micropachyiulus* Verhoeff, 1899. Apart from the unique lateral process, the opisthomere of *Bellatoiulus* gen. nov., unlike that in the aforementioned genera, has a fovea and lacks caudal lamella, the latter being well-developed in all three of them. The mesomeral process is well-differentiated in *Bellatoiulus* gen. nov. and *Geopachyiulus*, in contrast to *Micropachyiulus* and *Hylopachyiulus*, in which it is either poorly developed or mostly fused to the solenomere. Thus, there must be at least two groups of Pachyiulini genera that miniaturised independently of each other.

### ﻿Notes on the distribution and ecology

**Figure 10.**
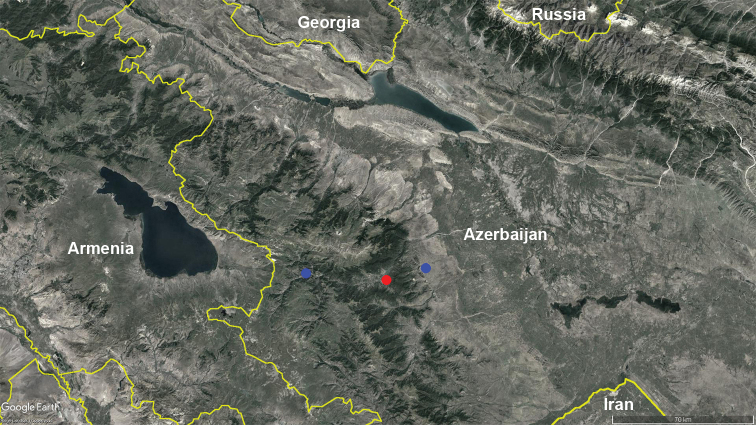
Distribution of *Bellatoiulusgolovatchi* gen. et sp. nov. Red circle: type locality; blue circles: records of addition material.

Apparently, *Bellatoiulusgolovatchi* gen. et sp. nov. is endemic to the eastern part of the Lesser Caucasus within Azerbaijan (Fig. 10), where it inhabits deciduous forests. However, future surveys may show that the species is also present in the adjacent territories of Armenia and Iran. According to the botanical biogeographic regionalisation of Menitsky (1991), the geographic distribution of the new species falls within the Murguz-Murovdag district of Eastern Transcaucasia.

Despite the absence of ommatidia and body pigmentation, *B.golovatchi* gen. et sp. nov. can hardly be regarded as an endogean or pedobiont, let alone a subterranean or hypogean form. Judging from both the present habitat information on the new species and personal observations on other blind (or with a strongly reduced number of ommatidia) and pale pachyiulinines, for example, species of *Apfelbeckiella* Verhoeff, 1901 or *Micropachyiulus* Verhoeff, 1899, *B.golovatchi* gen. et sp. nov. must be an inhabitant of the upper-most soil layer, immediately below the leaf litter. It could be thus assigned to the main life-form of Diplopoda–that of the stratobionts (Golovatch 1987), or alternatively to the hemiedaphic fauna (see e.g., Deltshev et al. 2011). By all means, the minute size and pale colouration combined with little or no surface activity and often patchy distribution within a confined geographic area make the “micropachyiulini” one of the least explored julids. Another study on a group of such genera distributed in the Balkans, the Southern Carpathians, and the Caucasus is currently in preparation (Vagalinski et al. in prep.).

## Supplementary Material

XML Treatment for Bellatoiulus

XML Treatment for Bellatoiulus golovatchi

## Supplementary materials

Supplementary material 1 *Bellatoiulusgolovatchi* gen. et sp. nov., male cybertype. Data type Video Explanation note Volume rendering of microCT image. https://binary.pensoft.net/file/674267This dataset is made available under the Open Database License (http://opendatacommons.org/licenses/odbl/1.0/). The Open Database License (ODbL) is a license agreement intended to allow users to freely share, modify, and use this Dataset while maintaining this same freedom for others, provided that the original source and author(s) are credited.Aleksandr P. Evsyukov, Boyan Vagalinski, Igor Y. Zabiyaka, Evgeniy V. Sadyrin

Supplementary material 2 *Bellatoiulusgolovatchi* gen. et sp. nov., female cybertype. Data type Video Explanation note Volume rendering of microCT image. https://binary.pensoft.net/file/674268This dataset is made available under the Open Database License (http://opendatacommons.org/licenses/odbl/1.0/). The Open Database License (ODbL) is a license agreement intended to allow users to freely share, modify, and use this Dataset while maintaining this same freedom for others, provided that the original source and author(s) are credited.Aleksandr P. Evsyukov, Boyan Vagalinski, Igor Y. Zabiyaka, Evgeniy V. Sadyrin
